# Biomarkers from in vivo molecular imaging of breast cancer: pretreatment ^18^F-FDG PET predicts patient prognosis, and pretreatment DWI-MR predicts response to neoadjuvant chemotherapy

**DOI:** 10.1007/s10334-017-0610-7

**Published:** 2017-02-28

**Authors:** Francesca Gallivanone, Marta Maria Panzeri, Carla Canevari, Claudio Losio, Luigi Gianolli, Francesco De Cobelli, Isabella Castiglioni

**Affiliations:** 1 Institute of Molecular Bioimaging and Physiology, National Research Council (IBFM-CNR), Via Fratelli Cervi 93, Segrate, 20090 Milan, Italy; 2 Department of Radiology, Centre for Experimental Imaging, San Raffaele Scientific Institute, Milan, Italy; 3 Department of Nuclear Medicine, Centre for Experimental Imaging, San Raffaele Scientific Institute, Milan, Italy; 4 Vita-Salute San Raffaele University, Milan, Italy

**Keywords:** Breast cancer, ^18^F-FDG PET/CT, DWI-MRI, Image features

## Abstract

**Objective:**

Human cancers display intra-tumor phenotypic heterogeneity and recent research has focused on developing image processing methods extracting imaging descriptors to characterize this heterogeneity. This work assesses the role of pretreatment ^18^F-FDG PET and DWI-MR with respect to the prognosis and prediction of neoadjuvant chemotherapy (NAC) outcomes when image features are used to characterize primitive lesions from breast cancer (BC).

**Materials and methods:**

A retrospective protocol included 38 adult women with biopsy-proven BC. Patients underwent a pre-therapy ^18^F-FDG PET/CT whole-body study and a pre-therapy breast multi-parametric MR study. Patients were then referred for NAC treatment and then for surgical resection, with an evaluation of the therapy response. Segmentation methods were developed in order to identify functional volumes both on ^18^F-FDG PET images and ADC maps. Macroscopic and histogram features were extracted from the defined functional volumes.

**Results:**

Our work demonstrates that macroscopic and histogram features from ^18^F-FDG PET are able to biologically characterize primitive BC, and define the prognosis. In addition, histogram features from ADC maps are able to predict the response to NAC.

**Conclusion:**

Our work suggests that pre-treatment ^18^F-FDG PET and pre-treatment DWI-MR provide useful complementary information for biological characterization and NAC response prediction in BC.

## Introduction

Evidence has shown that human cancers frequently display intra-tumor phenotypic heterogeneity [1, 2], whose nature can have profound implications both on tumor development and therapeutic outcomes. Measuring such heterogeneity could, therefore, be extremely useful in selecting the most promising therapeutic approach for an individual patient. Oncology is thus evolving in terms of the personalization of diagnostic and therapeutic clinical workup, where the key is the customization of practices tailored to individual patients.

Applying precision oncology to clinical practice is challenging. The translation of ex vivo omics technologies is an open issue due to the low availability of such platforms within hospitals, the high costs, limited accuracy and long time schedules required. Most of the procedures are also invasive and often require tissue sample extraction.

In vivo molecular imaging technologies, such as computerized tomography (CT), magnetic resonance (MR), functional diffusion-weighted imaging (DWI) MR, and positron emission tomography (PET) have acquired a new role in precision oncology. In addition to consolidating their role for diagnosis, staging, follow-up, and in guiding radiotherapy planning, they have shown interesting results in characterizing lesions, and in predicting prognosis and therapy response in many cancer diseases. This is particularly the case when quantitative indexes of tumor metabolism or function are used, such as tumor functional volume, apparent diffusion coefficient (ADC), standardized uptake value (SUV), or other derived indexes [3–5].

However, limited and contradictory results have been reported, and many authors have argued that such indexes are macroscopic features that are not always able to properly reflect the intra-tumor heterogeneity responsible for the different progression or therapy response of different phenotypes. To overcome these limitations, recent research has focused on developing advanced image processing methods that better extract imaging descriptors and better characterize such intra-tumor phenotypic heterogeneity. Several research groups have used textural indexes, extracting information on the intensity or on the shape and size of lesions, while others have considered indexes derived from wavelet analysis, with very promising findings [6–13].

In the case of breast cancer (BC), the role of MR and PET for diagnosis (including screening), initial staging, follow-up and response evaluation to therapy, detection of recurrences and guiding radiotherapy planning, was recently reviewed by Tabouret-Viaud et al. [14], on the basis of different clinical studies, mostly considering qualitative evaluations or quantitative assessments based on macroscopic image features and assessing the two imaging modalities, separately.

In this context, this work is intended to be an hypothesis-forming work, with the aim to assess the role of pretreatment ^18^F-FDG PET and DWI-MR with respect to BC prognosis and prediction of therapy outcome when image features are used to characterize BC primitive lesions. Both macroscopic and image features were extracted from ^18^F-FDG PET and DWI-MR images of BC primary tumors in a cohort of patients who had been referred for NAC before surgical resection. The relationship between the imaging features, histopathological/immunohistochemical indexes and clinical outcome is evaluated and discussed.

## Materials and methods

### Patients

A retrospective protocol, developed at the IRCCS San Raffaele Hospital, Milan, Italy, included adult females with biopsy-proven BC with BC diameter >1 cm as reported by mammography and referred for NAC treatment before surgical intervention. Patients were subjected to mammography and ultrasound for diagnostic purposes and to fine-needle biopsy in order to characterize the tumor. Subsequently, patients underwent a pre-therapy ^18^F-FDG PET/CT whole-body study for staging, and a pre-therapy breast multi-parametric MR study to complete primary BC staging. The protocol included T2-weighted sequences, DWI, and DCE using gadobutrol (Gadovist, Bayer Schering Pharma). Patients were then referred for NAC treatment and, at the end of the NAC, for the surgical resection of the tumoral bed or residual tumor. Surgically collected biological tumor samples were analyzed by a pathologist to determine the response to NAC. All patients provided their informed consensus for the whole protocol, including the collection of biological samples.

### In vivo molecular imaging studies

#### ^18^F-FDG PET

^18^F-FDG PET/CT studies were performed at the Nuclear Medicine Department-PET center of the IRCCS San Raffaele Hospital, on four different hybrid PET/CT scanners: a Discovery STE (General Electric Medical System), a Discovery ST (General Electric Medical System), a Discovery 690 (General Electric Medical System), and a Gemini TF (Philips Medical System).

Whole-body PET/CT studies were performed using the standard oncological protocol. Patients were injected with ^18^F-FDG (1 mCi/10 kg body weight), and the PET/CT exam was performed 60 min after the ^18^F-FDG injection. The ^18^F-FDG PET/CT oncological protocol included, a SCOUT scan at 40 mA, from the skull base of the patient to the middle thigh, a CT scan at 140 keV in helical mode, with an automatic modulation of the tube current in the range of 30–150 mA, during shallow breathing by patients and a ^18^F-FDG PET study, acquired in 3D mode (2.5 min/bed scan) for adjacent bed positions. PET images were corrected for attenuation, random and scatter noise component, and then reconstructed using a 3D ordered subset expectation maximization algorithm (OSEM). In order to provide an accurate quantification of the ^18^F-FDG uptake within the primary BC lesion, administered/residual ^18^F-FDG radioactivity and time of measurement were recorded after patient injection by a dose measurement system (Comecer). Patient weight and height were recorded for the same purpose.

### Multi-parametric MR

MR studies were performed at the Radiology Department of the IRCCS San Raffaele Hospital with a 1.5 T scanner (Achieva Nova, Philips Medical Systems), equipped with a dedicated double breast 7-channel coil (Breast Sense Coil).

MR sequences were acquired as follows. A T2-weighted Turbo Spin Echo study (TR 4000, TE 120, 436 × 323 matrix, 2.2-mm slice thickness, GAP 0.5, time of acquisition = 2′ 50″) was performed.

A DWI acquisition was performed using a single-shot echo-planar image (EPI) sequence on the axial plane with the following parameters: TR/TE 10,000/66 ms, FA 90°, matrix 224, field of view (FOV) 310 × 310, slice thickness 3 mm and acquisition time 70 s. Sensitizing diffusion gradients were applied along the *x*, *y,* and *z* axes with *b* value = 0 s/mm^2^ and *b* value = 900 s/mm^2^. ADC maps were generated using the imaging console, using the two DWI images with two different *b* value (*b* value = 900 s/mm^2^ and *b* value = 0 s/mm^2^) [15], applying the logarithmic function of the ratio between the signal intensity values (S) in the DWI images with different *b* values, as shown in the following equation

$${\text{ADC}} = \frac{{\ln \left( {\frac{{S_{0} }}{{S_{900} }}} \right)}}{{b_{900} - b_{0} }}.$$

A DCE study was acquired as follows. A T1-weighted Fast Field Echo sequence (TR: 499, TE: 4.6, 375 × 321 × 162 matrix, 2.5 mm slice thickness, FA 90°, GAP = 0, time of acquisition = 8′ 30″) was acquired, in order to obtain a pre-contrast map before the contrast injection. A dose of 0.1 mmol/kg of gadobutrol (Gadovist, Bayer Schering Pharma) was then injected (flow rate = 2 mL/s), followed by 20 mL of saline, and 10 s later, the same T1-weighted sequence was repeated five times. Following the acquisition, the pre-contrast T1 weighted images were subtracted from first post-contrast acquired images on a dedicated workstation (Viewforum, Philips), allowing obtaining subtraction images. Maximum intensity projection (MIP) reconstructions were applied to the subtraction images.

By using the AMIDE package [16], subtraction images were co-registered to DWI images (and, as a consequence, to the ADC maps), maintaining the slice thickness and matrix size of DWI images.

## Ex vivo histological and immunohistochemical studies

The biological samples were analyzed at the Pathological Anatomy laboratory of IRCCS San Raffaele Hospital.

The histological type and grade were evaluated from the samples collected from the core-needle biopsy. Tumors were classified according to the WHO guidelines [17]. A baseline immuno-histochemical characterization was also performed, evaluating the hormone receptor indexes (estrogen receptor—ER, and progesterone receptor—PR), the expression of c-erbB-2 oncoprotein, and the MiB-1 cellular proliferation index. Hormone receptor status was considered positive if an expression greater than 0% was found for ER or PR. Tumors were considered to over-express c-erbB-2 oncoprotein if more than 30% of invasive tumor cells showed definite membrane staining (Score 3+) or if a definite membrane staining was found smaller than 30% (Score 2+), resulting in a so-called fishnet [18].

Tumors were then classified into the various molecular subtypes including Luminal A, Luminal B, Triple negative/basal-like, and HER2 + [19]. Luminal A subtype included tumors showing a positive expression of ER and/or PR and a negative expression of c-erbB-2, while Luminal B subtype included tumors showing a positive expression of ER and/or PR and a positive expression of c-erbB-2. A tumor was classified as triple negative/basal-like if a negative expression of ER and PR together and a negative expression of c-erbB-2 were found. HER2 + subtype showed a negative expression of ER and PR and a positive expression of c-erbB-2 [20].

Response to NAC was assessed in the samples resected during surgery. Pathological complete response (pCR) was considered as achieved when absence of breast invasive cancer, irrespective of ductal carcinoma in situ or nodal involvement, was reported for the surgical specimens collected [21].

## Image features

### Features from ^18^F-FDG PET images

Different image features were extracted from PET images. Metabolic tumor volume (MTV) of the primary BC lesion was obtained using a fully automated image segmentation method, which combined an automatic threshold-based algorithm for the definition of MTV, and a k-means clustering algorithm for the estimation of the background. The method had been previously calibrated and validated on a variety of hybrid PET/CT scanners, including the PET/CT systems used in this work, and on a variety of oncological lesions, spherical/non-spherical, and/or with homogenous/non-homogenous ^18^F-FDG uptake, with an accuracy in the MTV measurement of 92% [22]. MTV was obtained as the number of PET voxels extracted by the segmentation algorithm multiplied by the voxel volume of the PET image.

Standardized uptake value (SUV). SUV of the primary BC lesion, weighted for the patient body weight, was calculated within the segmented MTV, according to the following formula [23]:

$${\text{SUV = }}\frac{{{\text{Tissue}}\;{\text{radioactivity}}\;{\text{concentration}}\;\,\left( {\frac{\text{MBq}}{\text{cc}}} \right)}}{{\frac{{{\text{Injected}}\;{\text{activity}}\; ( {\text{MBq)}}}}{{{\text{Patient}}\;{\text{body}}\;{\text{weight}}\; ( {\text{g)}}}}}}.$$

The mean and maximum intensities of SUV within the segmented MTV were calculated (SUV_mean_, SUV_max_). SUV_mean_ was corrected for the partial volume effect (PVE) by an automatic method, previously calibrated and validated on a variety of hybrid PET/CT scanners, including the PET/CT systems used in this work, and on a variety of oncological lesions, spherical/non-spherical and/or with homogenous/non-homogenous ^18^F-FDG uptake, ensuring a quantitative accuracy in the SUV_mean_ measurement of 93% [24–27].

Total Lesion Glycolysis (TLG) of the primary BC lesion was calculated as the product of SUV_mean_ (corrected for PVE) and MTV.

Fourteen features were extracted as first-order statistical features [13], which described the characteristics of the histogram of the PET voxel intensities within the segmented MTV.

Four shape- and size-based features were also extracted, which described the shape and size of the segmented MTV [13].

In summary, for all patients, a total of 22 PET imaging features were extracted for the primary BC lesions (see Table 1).

**Table 1:** Features extracted from PET and MR images

Type of feature	Imaging modality	Feature
Macroscopic features	PET	Metabolic tumor volume (MTV) [cc]
		Partial volume corrected mean body-weighted standardized uptake value (SUV_mean_) [g/cc]
		Maximum body-weighted standardized uptake value (SUV_max_) [g/cc]
		Total lesion glycolysis (TLG) [g]
	MR	Apparent diffusion coefficient functional volume (*V*_ADC_) [cc]
		Mean apparent diffusion coefficient (ADC_mean_) within *V*_ADC_ [mm^2^/s]
		Minimum apparent diffusion coefficient (ADC_min_) within *V*_ADC_ [mm^2^/s]
		Total lesion diffusion (TLD) [cm^5^/s]
Intensity-based features (first-order statistics)	PET/MR	Energy [(g/cc)^2^]–[(mm^2^/s)^2^]
		Entropy
		Kurtosis
		Maximum [g/cc]–[mm^2^/s]
		Mean [g/cc]–[mm^2^/s]
		Mean absolute deviation [g/cc]–[mm^2^/s]
		Median [g/cc]–[mm^2^/s]
		Minimum [g/cc]–[mm^2^/s]
		Range [g/cc]–[mm^2^/s]
		Root mean square (RMS) [g/cc]–[mm^2^/s]
		Skewness
		Standard deviation [g/cc]–[mm^2^/s]
		Uniformity
		Variance[(g/cc)^2^]–[(mm^2^/s)^2^]
Shape- and size-based features	PET/MR	Surface area [cm^2^]
		Spherical disproportion
		Sphericity
		Surface to volume ratio [cm^−1^]

### Features from DWI-MR images

For each BC lesion, different image features were extracted from the MR ADC map.

A three-dimensional (3D) functional volume was defined on the ADC maps (*V*_ADC_) by an adaptation of the segmentation method developed by Nan-Jie Gong et al. [28], but taking advantage from the availability of the DCE acquisition to eliminate the need for manual lesion annotations. Nan-Jie Gong et al. [28] proposed the measure of the lesion functional volume by overlapping the parts of lesion tissue obtained from DWI images with *b* value = 0 s/mm2 (DWI b0 image) and ADC maps, after classifying and excluding probable non-carcinoma compartments from both the images. In their procedure, taking ADC maps as references and on DWI b0 images, ROIs were drawn manually along the lesion contour by radiologists and used as input for segmentation. Our procedure is based on the same approach for excluding probable non-carcinoma compartments but is a semi-automated procedure, improving the efficiency of volume estimation, implemented with homemade software.

**Fig. 1:**
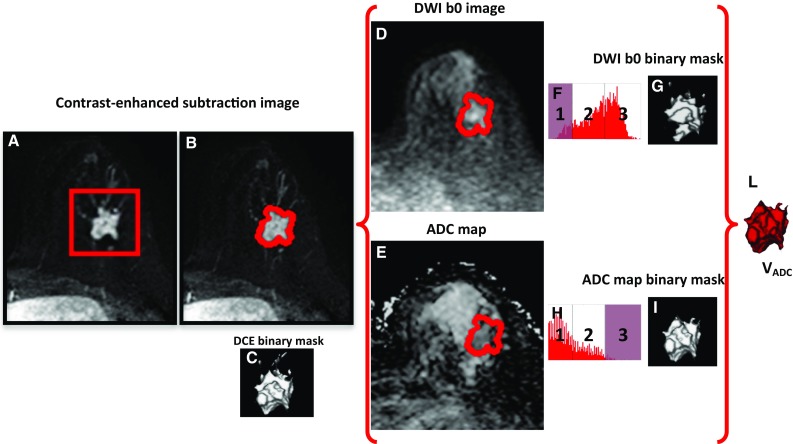
Procedure for the semi-automated extraction of the lesion functional volume from MR ADC maps. **a** The user draws a *3D rectangular box* on the lesion on DCE subtraction images; **b** the enhanced tissue is segmented; **c** the DCE binary mask of the enhanced tissue is generated; **d** the DCE binary mask is applied on DWI b0 images; **e** the DCE binary mask is applied on the ADC maps; **f** a three compartment k-means algorithm is applied on signal intensity to classify DWI b0 images; **g** the DWI b0 binary masks is generated by excluding low signal (compartment 1—noise, fat, and fibrous tissue); **h** a three compartment k-means algorithm is applied on signal intensity to classify ADC maps; **i** the ADC binary masks is generated by excluding high signal (compartment 3—cyst, necrosis, fluid, normal tissue, and noise); **l** the probable spatial extension of high cellularity *V*_ADC_ is the overlap between the DWI b0 and the ADC binary masks

The different steps involved in segmentation procedure are shown in Fig. 1.

As a preliminary step, the operator is required to define a 3D rectangular box on the DCE subtraction images, including the BC lesion (see Fig. 1a). Using a two compartment k-means algorithm, the segmentation of enhanced tissue is automatically obtained (see Fig. 1b) and a binary mask of the enhanced tissue is then generated (see Fig. 1c). This step allows locating and limiting, in a semi-automatic way, the spatial extension for lesion signal segmentation on the DWI b0 images and ADC maps, thus improving the efficiency of volume estimation and avoiding inter-observer variability with respect to the manual delineation on DWI b0 images proposed by Nan-Jie Gong et al. [28]. The extracted binary mask is applied to both DWI b0 images and ADC maps, thus defining the enhanced lesion on these images (see Fig. 1d, e).

A three compartment k-means algorithm is then applied to the DWI b0 images, allowing classification of signal into three classes: (1) noise, fat, and fibrous tissue, showing low signal on DWI b0 images, (2) high-cellularity tumor tissue, showing an intermediate signal on DWI b0 images, and (3) cyst, necrosis, fluid, and normal tissue, showing high signal on DWI b0 images. The cut-off point between low signal and intermediate signal, automatically obtained by the k-means procedure, is considered as lower cut-off point for DWI b0 images (see Fig. 1f). Using this low threshold, a binary mask is generated from DWI b0 images excluding low signal (compartment 1), and including only signal belonging to compartments 2 and 3 (see Fig. 1g).

The three compartment k-means algorithm is then applied to the ADC maps, allowing classification of signal into three classes: (1) Fat and fibrous tissue, showing low ADC signal, (2) high-cellularity tumor tissue, showing intermediate ADC signal and (3) cyst, necrosis, fluid, normal tissue and noise, showing high ADC signal. The cut-off point between high and intermediate ADC values, automatically obtained by the k-means procedure, is considered as higher cut-off point for ADC maps (see Fig. 1h). Using high threshold, a binary mask is generated from ADC maps excluding high signal (compartment 3), and including only signal belonging to compartments 1 and 2 (see Fig. 1i).

The overlap between the final DWI b0 binary mask and the ADC binary mask represents the probable spatial extension of ADC functional volume (*V*_ADC_), with the exclusion of non-tumor tissues (see Fig. 1l).

Mean ADC (ADC_mean_) and minimum ADC (ADC_min_) of the primary BC lesion were obtained as mean and minimum values of diffusion coefficients measured within the segmented *V*_ADC_. In analogy with the TLG defined in PET, we defined total lesion diffusion (TLD) calculated as the product between ADC_mean_ and *V*_ADC_, as a functional index reflecting diffusion integrated on the functional ADC volume.

Fourteen features were extracted as first order statistical features [13], which described the characteristics of ADC map voxel intensities within the segmented *V*_ADC_. Four shape and size based features were also extracted, thus describing the shape and size of the segmented *V*_ADC_ [13].

In summary, a total of 22 MR imaging features were extracted for the primary BC lesion (see Table 1).

## Statistical analysis

A statistical analysis was performed to assess the relationship between (1) PET and MR features, (2) PET or MR features and histopathological and immuno-histochemical parameters, and 3) PET or MR features and response to NAC as defined by pCR. Different statistical tests were used. Spearman correlation was used to evaluate the relationships between each macroscopic PET parameter and each macroscopic RM parameters (SUV_mean_ vs. ADC_mean_; SUV_max_ vs. ADC_min_; MTV vs. *V*_ADC_; TLG vs. TLD), as well as between each PET feature vs. each corresponding MR feature. Linear regression was applied to evaluate the form of imaging biomarkers relationship, in particular for functional volumes (*V*_ADC_ vs. MTV). Relationships between PET and RM imaging biomarkers with histological and immunohistochemical data was assessed using Mann–Whitney and Kruskal–Wallis tests. In particular Kruskal–Wallis test was used when patient’s data were categorized in more than two classes following ex vivo data (i.e. in case of analysis vs. molecular subtypes). In performing statistical tests no correction was applied for accounting for multiple comparison, since our study was an explorative study on a small cohort of patients with the aim to form hypothesis on the potential role of accurate functional quantitative imaging biomarkers from PET and MRI for BC characterization and evaluation of response to chemotherapy, to be validated on a larger cohort.

**Fig. 2:**
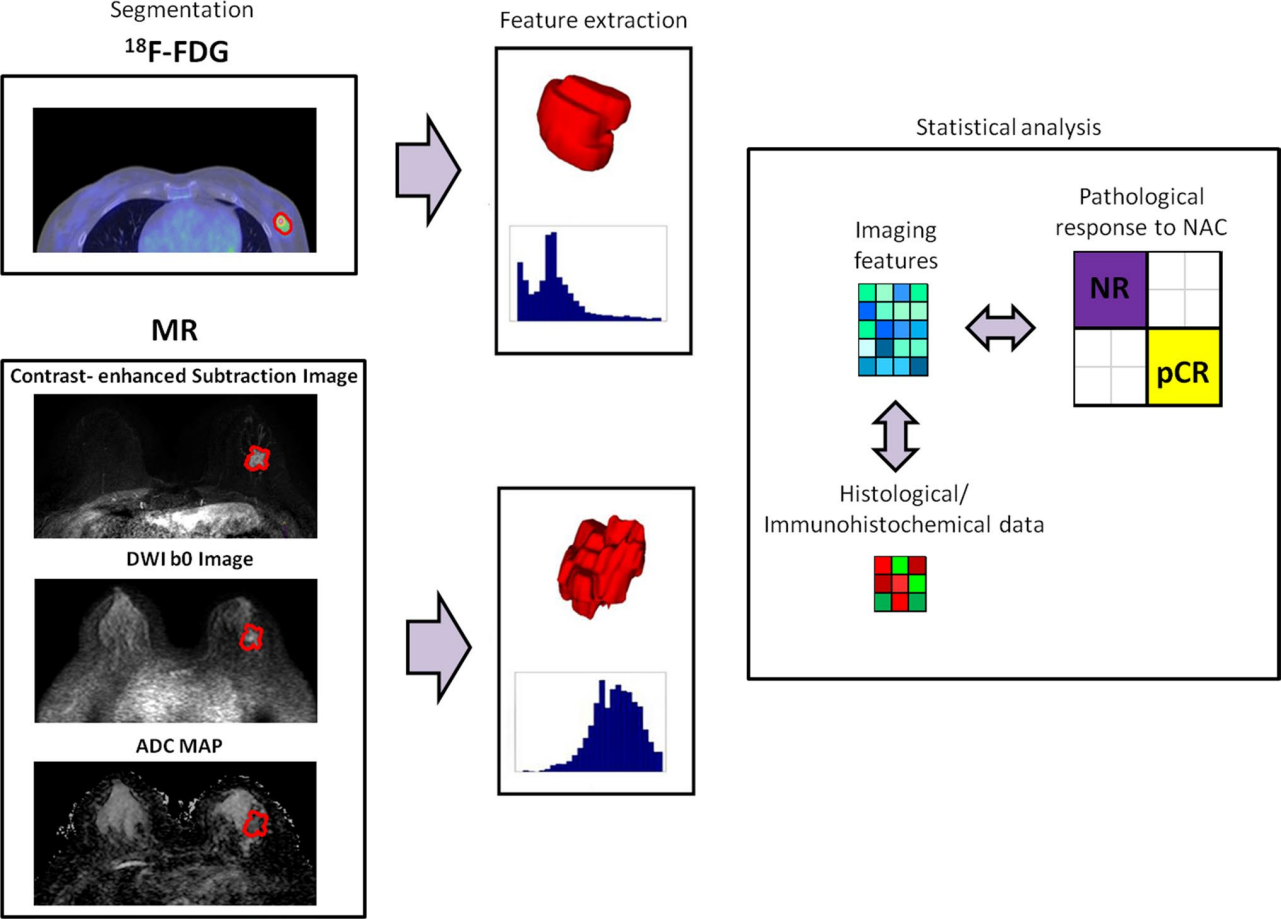
Working strategy: image segmentation, features extraction, and statistical analysis

Figure 2 shows the working strategy including image segmentation, feature extraction, and statistical analysis.

## Results

### Patients

The retrospective protocol included 38 adult females (age range 28–72 years; average age = 48 ± 12 years). All patients underwent ^18^F-FDG PET, multi-parametric MR studies, and histological/immunohistochemical analyses. Only 31/38 (82%) of patients underwent surgery at the end of NAC. The average time interval between pretreatment ^18^F-FDG PET/CT and pretreatment multi-parametric MR was 10 ± 10 days.

### In vivo molecular imaging data

#### In vivo ^18^F-FDG PET

^18^F-FDG PET/CT images were interpreted by an experienced nuclear medicine physician. ^18^F-FDG PET/CT studies detected the primary BC lesion in 100% of the considered patients. Qualitative analysis of ^18^F-FDG PET/CT images enabled the PET lymph node status to be defined. A positive PET lymph node status was reported when the PET/CT exam showed ^18^F-FDG uptake in at least one of the patient’s lymph nodes. A total of 57% of the patients were PET lymph node positive, and 43% were PET lymph node negative.

#### Multi-parametric MR

MR images were interpreted by two experienced radiologists (CL and MP, with 20 and 5 years of experience in breast MRI, respectively). MR studies detected the primary BC lesion in 100% of the patients. The T2-weighted images revealed the presence of intratumoral necrosis, pseudocapsule and peritumoral/prepectoral edema. Intramural necrosis was present in 13 patients (34%), pseudocapsule in 10 (26%), and peritumoral edema in 16 (42%). The late subtraction images revealed rim enhancement in 23 patients (60%). An asymmetrical development of breast vascular maps due to the presence of tumor was found on MIP reconstruction in 63 women (70%). Lymph nodes were not considered in the MR evaluation.

### Ex vivo histological and immunohistochemical data

Tumors were characterized by fine-needle biopsy. All the patients (100%) were diagnosed with IDC. Biopsy did not define the histological grade in 11 patients (29%). The histological grade of the remaining 27 patients was distributed as follows: 22% had a G2 tumor, and 78% of patients had a G3 tumor.

A total of 47% of patients were found to be ER positive, while the remaining 53% were ER negative. Thirty-nine percent of patients were PR positive, while the remaining 61% were PR negative. A negative expression of c-erbB-2 was found in 79%, while the remaining 21% of tumors showed a positive expression of c-erbB-2.

MiB-1 proliferation index was quantified ≥18% in 81% of the tumors. This cut-off was used as a threshold for positive staining based on the mean values obtained in the histopathology laboratory.

The distribution in molecular subtypes was represented as follow: 24% of tumors were classified as the Luminal A subtype, 21% as Luminal B, 13% as HER2 + subtype, 42% as the triple negative/basal-like subtype. The percentage of triple-negative/basal-like subtype can appear to be high, but this is because, in our institution, the clinical workup of patients diagnosed with breast cancer is not standardized for radiological examination and that the patients, which are addressed to both a pre-therpy PET syudy and to a pre-therapy MRI study, are mainly the triple-negative patients.

For patients undergoing surgery at the end of NAC (31), pathological pCR was investigated. A total of 42% of patients showed a complete response to NAC treatment, while the remaining 58% were classified as non-complete responders. Normally only about 20–30% patients respond completely, in the patient cohort considered in this study there is a high presence of triple negative patients thus justifying these percentages [29].

Table 2 shows the results of the indexes measured from histological/immunohistochemical analyses.

**Table 2:** Histological/immunohistochemical data for the patient population

Histopathological index		Frequency (%)	Mean ± *δ* (%)	Range (%)
Histological type	IDC	100		
Histological grade	G1	3		
	G2	13		
	G3	55		
	G not available	29		
ER	Positive (>0%)	47	71 ± 31	0–90
	Negative (<0%)	53	0 ± 0	
PR	Positive (>0%)	39	38 ± 38	0–80
	Negative (<0%)	61	0 ± 0	
c-erbB-2	Positive (Score 3+/Score 2+ FISH positive)	21		
	Negative	79		
Molecular subtype	Luminal A	24		
	Luminal B	21		
	Triple Negative/basal-like	42		
	HER2+	13		
MiB-1	Positive (≥18%)	81	44 ± 15	4–80
	Negative (<18%)	19	15 ± 4	

### Image features

#### Features from ^18^F-FDG PET images

The mean MTV value of the BC lesions, as measured on the PET images, was 18.76 ± 40.61 cc (sphere-equivalent diameter = 1.65 ± 2.13 cm, within a range of 0.61–3.88 cm).

SUV_BW-mean_ was measured for 100% of primary BC lesions, with a mean value of 11.36 ± 8.92 g/cc within a range of 1.47–40.25 g/cc.

SUV_BW-max_ was measured for 100% of primary BC lesions, with a mean value of 12.16 ± 10.39 g/cc within a range of 2.01–36.98 g/cc.

TLG was measured for 100% of primary BC lesions, with a mean value of 268.92 ± 585.89 g/cc within a range of 2.0–2602.25 g/cc.

Imaging features were extracted from PET images in all the patients.

#### Features from multi-parametric MR images

*V*_ADC_ was successfully extracted semi-automatically for all patients. All the lesions showed an enhancement in the DCE acquisition which was captured by our algorithm, thus confirming the possibility of performing an initial automatic segmentation on DCE acquisition in order to fully automate the procedure for measuring the lesion volume.

The mean value of *V*_ADC_ of the BC lesions, as measured on the ADC maps, was 17.18 ± 28.26 cc (sphere-equivalent diameter = 1.60 ± 1.89 cm, within a range of 0.46–3.24 cm).

ADC_mean_ was measured for 100% of primary BC lesions, with a mean value of 1102 ± 422 mm^2^/s, within a range of 480–1951 mm^2^/s.

ADC_min_ was measured for 100% of primary BC lesions, with a mean value of 151 ± 159 mm^2^/s within a range of 7–666 mm^2^/s.

TLD had a mean value of 213.93 ± 455.71 cm^5^/s, within a range of 4.24–2640.56 cm^5^/s.

Imaging features were extracted from MR images in all the patients.

### Statistical analysis

#### PET vs MR features

**Fig. 3:**
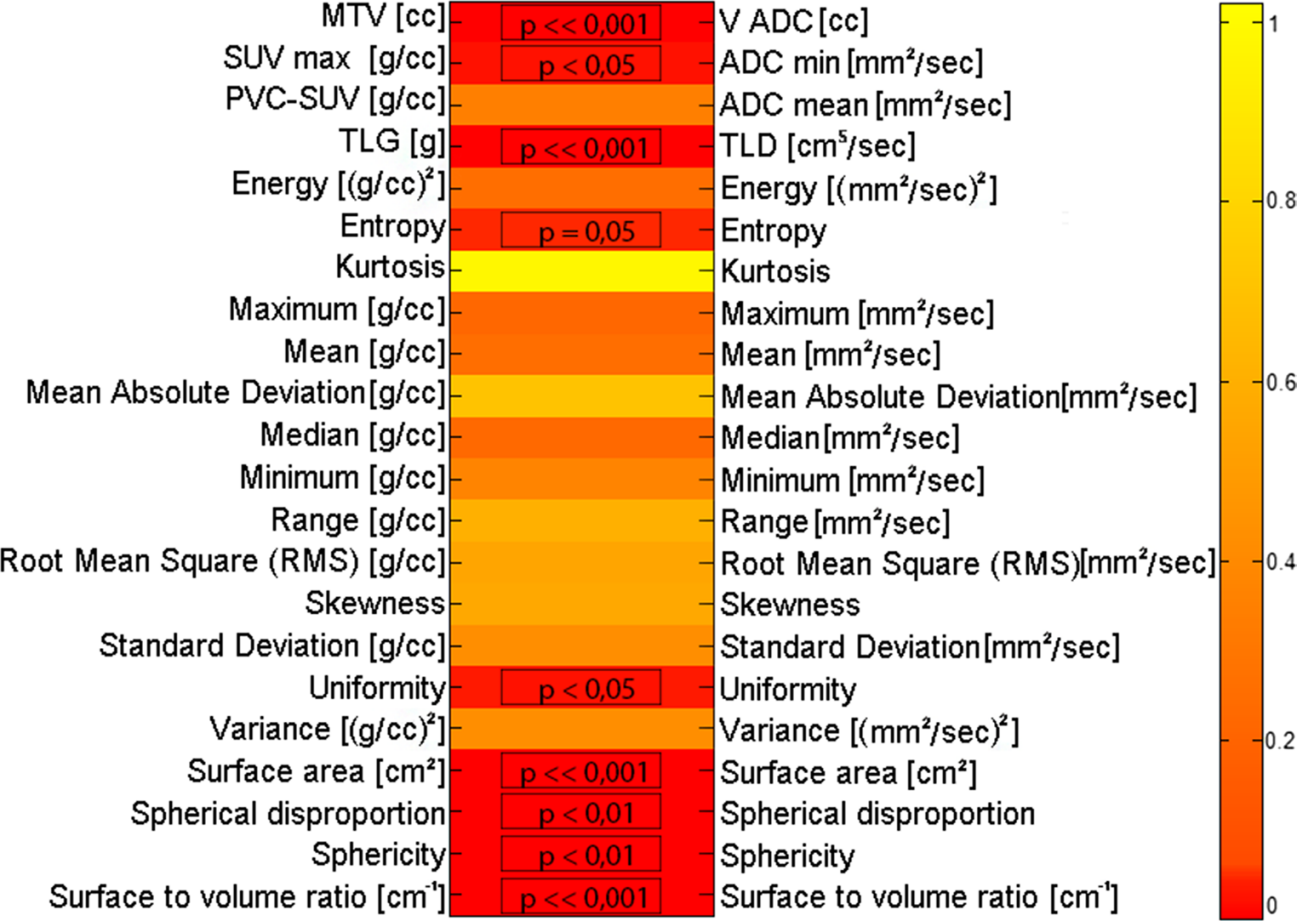
Statistical correlations between PET and MR macroscopic and textural features (*p* values from Spearman correlation test)

The results of the statistical correlations between PET and MR macroscopic and textural features are shown in Fig. 3.

There are strong correlations between the macroscopic features MTV and *V*_ADC_ (*p* value ≪0.001, Spearman test), and TLG and TLD (*p* value ≪0.001, Spearman test). Significant correlations are also evident among all the shape-and-size based (*p* < 0.01, Spearman test). More specifically, linear regression confirms a positive linear relationship between the two functional volumes (correlation coefficient = 0.86; *V*_ADC_ = a*MTV + *b*; *a* coefficient = 1.17, *b* coefficient = 0.06663; *R* ^2^ = 0.72).

Considering the features representing the intensity of the functional signal within the tumor, significant correlations were found only among SUV_max_ and ADC_min_ (*p* value <0.05, Spearman test), and among the PET histogram features “Uniformity” and “Entropy” with the corresponding histogram features as measured by DWI-MR (*p* value <0.05, Spearman test). Linear regression showed that there is a negative relationship between SUV_max_ and ADC_min_, also with a low significance (correlation coefficient = −0.3, *R* ^2^ = 0.14), thus showing that SUV_max_ and ADC_min_ are surrogate biomarkers of different and inversely correlated functions.

#### PET and MR features vs. histopathological/immunohistochemical parameters

Given that 100% of patients had IDC, the histological grade was not available for 29% of patients, and only 21% of patients were positive for c-erbB-2, correlation analyses were performed only with hormone ER and PR receptor status, with molecular subtype and with MiB-1 proliferation index. The MiB-1 proliferation index was considered as a continuous variable due to the unbalanced sample of patients with positive Mib-1 proliferation index (81%). With regard to the molecular subtype, patients were analyzed using the classification into the four molecular subtypes (all), but also by re-arranging them into just two classes (Triple-Negative tumors vs. Non-Triple-Negative tumors), in order to have more balanced and larger classes for the statistical analyses.

**Fig. 4:**
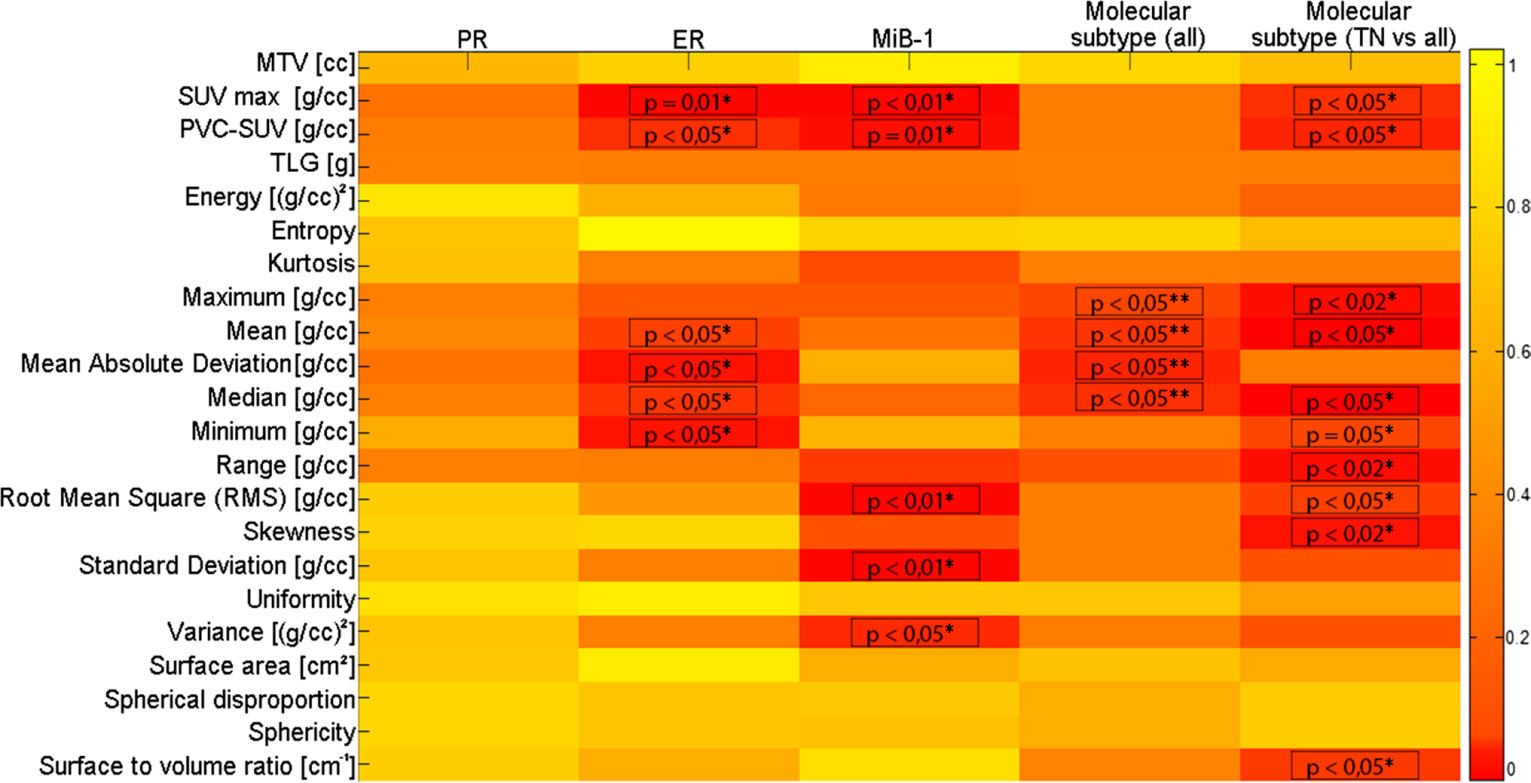
Statistical correlations between PET textural features and histological/immunohistochemical data (* = *p* values obtained by Mann–Whitney test, **p values obtained by Kruskal–Wallis test)

The results of the statistical correlations between PET textural features and histological/immunohistochemical data are reported in Fig. 4.

The geometrical texture “surface to volume ratio” is clearly the only geometrical feature with a biological role in this analysis. This suggests that a biological characterization of BC tumors is better performed by PET image features representing the intensity rather than the geometry of the functional uptake of a BC lesion.

With regard to the hormone receptor status, no statistically significant correlation was found between PET features and PR expression, while both SUV_max_ and PVC-SUV_mean_ were found to be significantly correlated to ER expression. Also many histogram-based features were found to be significantly correlated to ER expression, supporting the results obtained by the macroscopic features directly describing the intensity of the functional uptake of the tumor (SUV_max_ and PVC-SUV_mean_).

The macroscopic features PVC-SUV_mean_ and SUV_max_ showed significant correlations with MiB-1, thus confirming previous published results on their role as surrogate biomarkers of proliferation [26, 30]. Also, some intensity-based textural features were found to be correlated with MiB-1, supporting the results obtained by the macroscopic intensity-based features PVC-SUV_mean_ and SUV_max_.

An interesting result was obtained by examining the distribution of PET features in the four classes of molecular subtypes. While no correlations were found considering the PET macroscopic features, different histogram-based features were found to be significantly correlated with molecular subtypes, thus showing a superior ability to characterize the tumor, compared to macroscopic features. TN tumors have a more aggressive phenotype with respect to the other molecular subtypes due to their higher proliferation and invasiveness. Because of these characteristics, different research efforts are dedicated to develop therapeutic strategies specific for TN tumors [31]. With the purpose to evaluate if PET imaging biomarkers are able to selectively characterize TN tumors, we performed also the comparison of PET imaging features in TN tumors vs. the group of other molecular subtypes. In our results, SUV_max_ and PVC-SUV_mean_ , as well as several imaging features were found to be significantly different in TN tumors than in the other subtypes. These results suggest that PET could have a role in the characterization of TN forms. Furthermore, since the PET features considered are correlated to prognostic factors in BC (e.g. Mib-1), these results suggest that PET could play a particular role in the prognosis of BC.

**Fig. 5:**
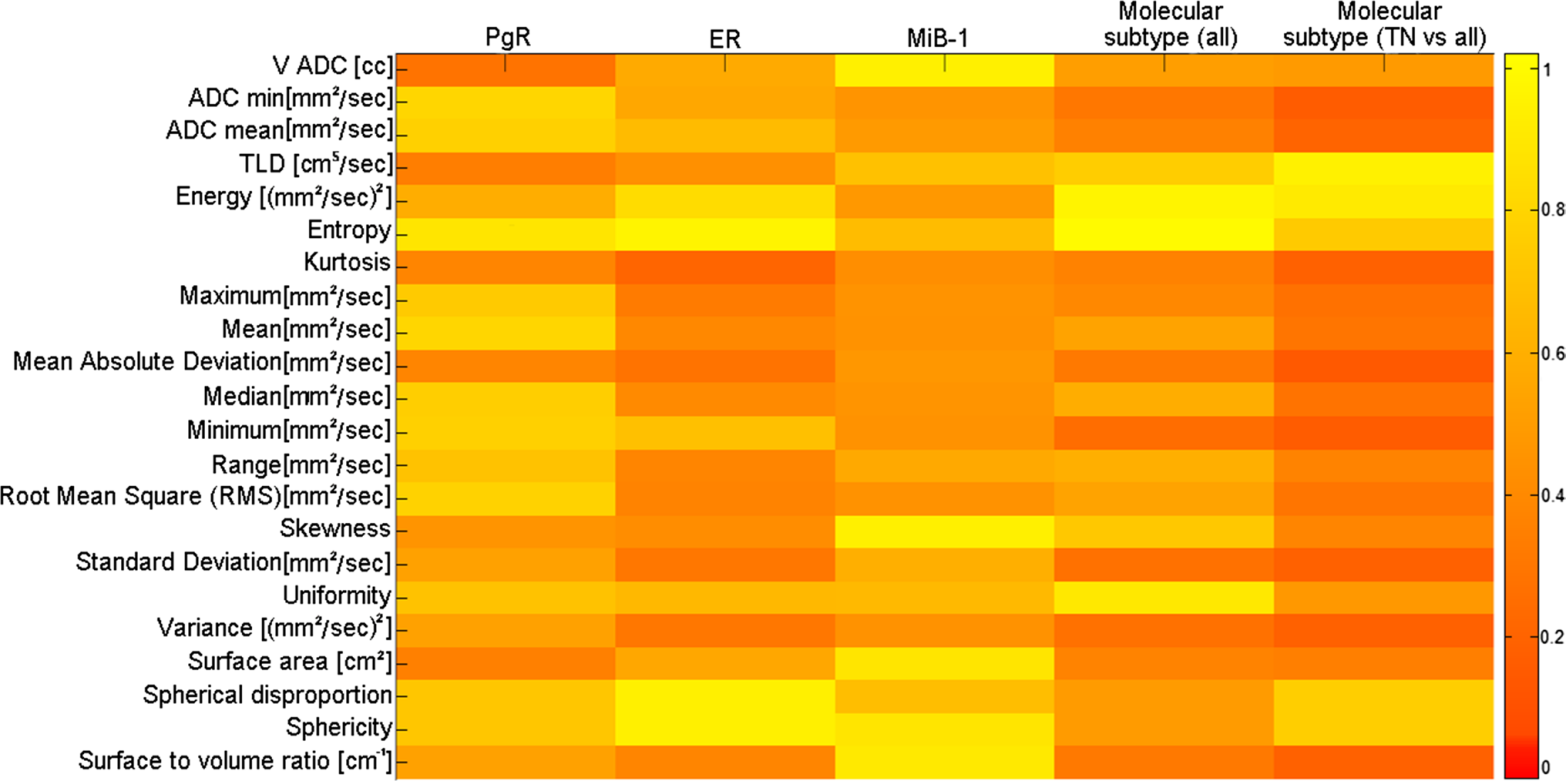
Statistical correlations among ADC map textural features and histological/immunohistochemical data (* = *p* values obtained by Mann–Whitney test, **p values obtained by Kruskal–Wallis test)

The results of the statistical correlations between DWI-MR textural features and histopathological/immunohistochemical data are reported in Fig. 5. None of the MR features (either macroscopic or textural) were found to be significantly correlated with any histological/immunohistochemical data.

#### PET and MR features vs pCR

**Fig. 6:**
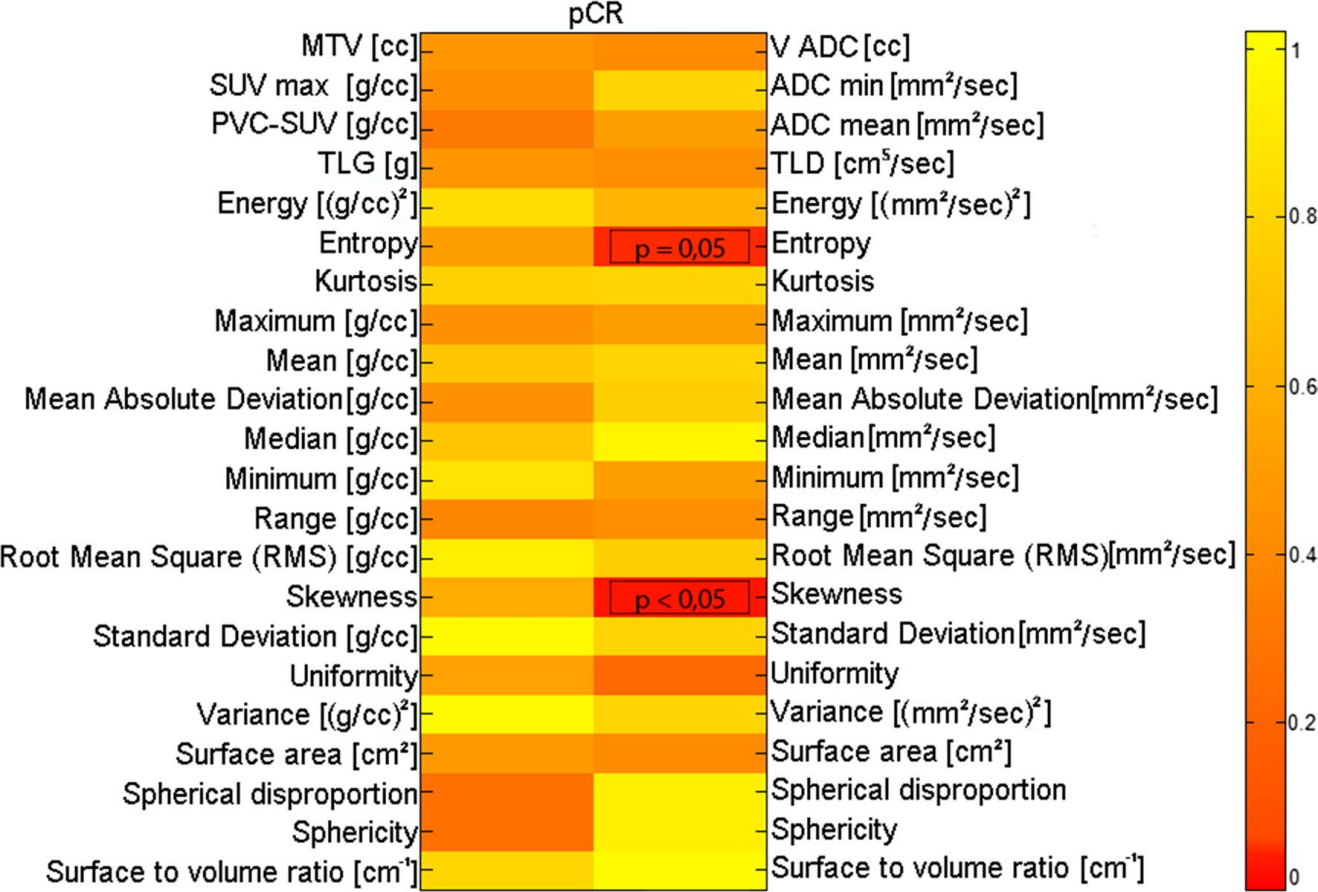
Statistical correlations between PET (*left*) and MR (*right*) image features and pCR (*p* values obtained by the Mann–Whitney test)

The results of the statistical correlations between PET features and pCR are reported in Fig. 6. None of the PET features (either macroscopic or textural) were found to be significantly correlated with pCR to NAC. This suggests that PET has no role in the prediction of NAC treatment.

The results of the statistical correlations between MR features and pCR are reported in Fig. 6.

The histogram-based features “Entropy” and “Skewness” were found to be significantly correlated with pCR (*p* value = 0.05 and *p* value <0.05, respectively, Spearman test). These results are of particular interest because they suggest two features linked to the heterogeneity of diffusion within the BC lesion as surrogate biomarkers to predict response to NAC.

## Discussion

MR and PET are acquiring a new role in studies on BC, in particular when primitive tumor images are quantitatively assessed. Considering ^18^F-FDG PET/CT, several works have been published on quantitative macroscopic features, such as MTV, SUV, or TLG, extracted from PET images of BC and validated as surrogate biomarkers for the biological characterization of BC, as well as for the prognosis and prediction of BC patients’ therapy responses. However, the results obtained by the various studies are controversial due to several factors limiting the accuracy of response evaluation by ^18^F-FDG PET/CT, such as the low pretreatment uptake of some tumors (e.g. in invasive lobular carcinoma and low-grade tumors) [32, 33]. More recent studies have focused on extracting textural features from ^18^F-FDG PET/CT images and evaluating the role of such advanced indexes in BC [34, 35]. These studies underline the potential of this analysis in the context of BC biological characterization, showing the capability of ^18^F-FDG PET/CT textural indexes to be independent predictors of invasive components [34]. In addition, they show that tumor heterogeneity measured on FDG-PET/CT is higher in invasive breast cancer with poor prognosis pathological factors [35]. However, exactly which histological or biological factors lead to differences in tumor features as measured by 18F-FDG PET/CT images remains unclear.

With regard to MR, fewer works have been published with respect to PET on the quantification of features from DWI-MR images. Furthermore, texture analysis has been applied to multi-parametric MR images in a very limited number of works and, to our knowledge, only in one work in BC, in a preclinical study [36].

Different methodological issues are still open on the image processing of ADC maps. There is a great variety both in the methods to process ADC maps and in the methods to extract ADC-derived biomarkers. Most studies use manually derived regions of interest, which is limited due to the operator-dependent approach, or small fixed regions manually placed within detected lesion and does not evaluate the accuracy and reproducibility of such procedures in the definition of potentially useful indexes for the prognosis or prediction of therapy responses. Furthermore in most of the work, mean ADC is defined only on a two-dimensional defined region, excluding the inherent 3D spatial distribution of ADC signal, corresponding to the 3D lesion shape. In defining quantitative ADC-derived parameters, different studies do not deal with the separation of contribution of noise, necrotic or cystic tissue within the lesions, thus giving ADC values which may not reflect only proliferative cancer tissue. However, it is acknowledged by physicists, radiologists and oncologists that the different tissues could contribute differently to DWI b0 and AD maps signal intensities [37, 38], thus potentially giving the ability to distinguish the different contribution by image classification [28].

In this limited scenario, our work evaluates the role of ^18^F-FDG PET/CT and DWI-MR in BC when both macroscopic and imaging features (from histogram and with shape and size descriptors) are extracted for prognosis of BC and prediction of the response to NAC.

We believe that our work represents an advance both in terms of methodologies and clinical results. Our segmentation method segments the MR functional volume of lesions on the ADC map (*V*_ADC_) which, in BC, is directly correlated to the PET metabolic volume MTV. Regarding MR, most works have assessed the role of ADC as surrogate biomarkers for characterization and treatment response prediction [39]. To our knowledge, only one study has proposed and validated a methodology to segment ADC maps and to obtain volume-like ADC parameters as candidate biomarkers for diagnosis and prognosis [28]. That study adopted a conservative approach based on the exclusion of non-carcinoma compartments from the segmented lesion volume rather than the direct classification of high-cellularity tumor tissues. Although histological verification was not available in [28], the radiologists visually validated the final segmented tissue as high-cellularity tumor tissue according to the available PET/CT and DW MR images. In our work we used the same conservative validated methodology and we implemented a semi-automated procedure to obtain an ADC functional volume. Taking advantage of the availability of DCE images, we eliminated the need of manual contouring of lesions from radiologists as input to the segmentation procedure, as required in [28]. Then, within the defined ADC functional volume, we extracted both macroscopic and advanced MR features. The linear relationship between the PET functional volume and the volume obtained by our methodology on ADC maps is an indirect result of the effectiveness of our procedure.

We defined a new parameter that characterizes the MR total diffusion of lesions (TLD), which, in BC, results directly correlated to TLG from PET. TLD was defined as the product between ADC and *V*_ADC_, similarly to the PET index TLG which is defined as the product between SUV and MTV. TLG was introduced in order to evaluate changes in functional glucose uptake by reflecting total lesion glycolysis within the metabolic volume, irrespectively of any changes in metabolic volume [40]. This functional index has been extensively validated as a PET biomarker for the diagnosis, prognosis and prediction of therapy in several cancer diseases [3, 41–44]. The significant direct correlation between TLD and TLG found in our work suggests that the total lesion diffusion within that part of volume showing an altered diffusion could be considered as functional parameter reflecting altered biological processes as those occurring in cancer tissue. We believe that this new parameter could be important in relationship with its variation during therapy, when functional RM imaging is available at different patient evaluations, for example during NAC.

We found an inverse correlation between SUV_max_ and ADC_min_. Studies on the relationship between SUV and ADC have produced conflicting results [4, 39, 45]. When a negative correlation between these two parameters was found, it was suggested as highlighting the biological relationship between glucose metabolism and diffusion within a lesion [45]. Our investigations, as well as other studies [46, 47], showed negative correlations between these two biomarkers, even though with a low significance.

PET and MR intensity-based features were found to be poorly or not correlated. With the exception of “Uniformity”, no other intensity feature was found to be correlated as measured by FDG-PET and DWI-MR. This suggests that the two imaging techniques are able to evaluate the tumor signal heterogeneity in a different way.

PET and MR shape- and size-based features were found to be strongly correlated, suggesting that the two imaging techniques are able to reveal tumor geometric characteristics in a similar way.

We found that some of the PET features are biomarkers for prognosis, in fact features representing the intensity of the metabolic uptake of the tumor at a macroscopic or histogram level were found to be correlated with biological prognostic indexes. Specifically, SUV_max_ and PVC-SUV_mean_ were correlated with ER, Mib-1, and TN molecular subtype and intensity-based histogram features were correlated with ER and molecular subtype. With the exception of “surface-to-volume ratio”, the other features related to the geometrical characteristics of the tumor, at both a macroscopic (MTV, *V*_ADC_, TLG, TLD) and a voxel-level (shape- and size-based festures) were not found to have a role in prognosis. Our results are in agreement with those obtained by Soussan M et al. [35], showing that PET textural features have a prognostic role in BC and that the cell metabolism heterogeneity and are factors related to BC characteristics.

Considering DWI-MR, some DWI-MR features are biomarkers for predicting the response to NAC. Features representing the heterogeneity of the diffusion intensity of the tumor at a histogram level—specifically, “Entropy” and “Skewness”—were found to be correlated with the response to NAC. The features related to the geometrical characteristics of the tumor, at both macroscopic and histogram levels, were not found to have a role in predicting the response to NAC. As reported in a recent review by Leong KM et al. [48], conflicting results were obtained when considering the role of ADC at the baseline in predicting the response to NAC. Furthermore, to our knowledge, no studies have investigated the features at the voxel level from ADC maps vs. pCR. Our results suggest a potential role of baseline DWI-MR in predicting pCR to NAC.

We found a different and complementary role of PET and DWI-MR in BC; in fact, in our study the pretreatment ^18^F-FDG PET was able to predict patient prognosis, while pretreatment DWI-MR managed to predict the response to NAC. Our results are of particular interest considering the ongoing spread of new generation hybrid PET/MR scanners which could be used as a single imaging examination for theranostic purposes in BC.

However, our work presents several limitations. We considered a retrospective protocol focused on invasive BC, thus including only one type of BC and with a limited sample size of 38 patients. This limited the possibility to fully exploit the relationship between image descriptors and breast tumor biological features.

Although we found several significant results by using univariate analyses, a sample size of 38 patients was not sufficient to extend our results to clinical practice. Furthermore, we did not consider conservative approaches in terms of statistical thresholds that prevent the possibility of false positive findings due to multiple comparison. Our results should be thus confirmed using larger cohorts using more conservative statistical threshold corrected for multiple comparisons and studying the impact of advanced imaging quantitative analysis on other BC subtypes. Furthermore, on larger cohorts, multivariate approaches could be used to see how a combination of PET and MR features is able in predicting pathology response, and outcome.

Other improvement could be achieved including other parameters in the model, for example pharmacokinetics parameters in the MR model obtained from DCE MR images, or considering the impact of other portions of the tumors, e.g. the necrotic tissue, since the treatment response could reveal differences between viable and necrotic portions.

Even if the algorithm for the extraction of *V*_ADC_ was found clinically feasible since all the considered images were successfully segmented, a validation of the segmentation algorithm was not performed with respect to an accurate gold standard or in comparison with other segmentation procedures. Trained radiologists should approve our *V*_ADC_ segmentation, and preferably compare it against manual segmentations to assess its accuracy. The method may be also potentially optimized to extract directly high-cellularity tissue compartment on b0 or ADC maps.

Furthermore, our work is based only on image indexes obtained from the histogram of the voxel values within the tumor, the heterogeneity descriptors (HDs) disregard the inherent spatial relationship between voxel values, only reflecting the voxel-value frequency distribution, thus including only first-order statistical indexes. However, alternative approaches account for the spatial arrangement of voxel values within the tumor using higher-order statistics by first calculating a 2- or 3-dimensional matrix describing this spatial organization. This approach may also be more informative than heterogeneity image characteristics. In this direction, more detailed studies should focus on the use of higher-order statistical descriptors, also for examining the relationship between the various image descriptors that describe “heterogeneity” [49].

## Conclusions

Our work demonstrates that textural features extracted from pre-treatment ^18^F-FDG PET and DWI-MR are able to define patient prognosis and to predict response to NAC in BC. Our results are of particular interest in terms of potential exploitation with hybrid PET/MR scanners, which could be used as a single imaging examination for theranostic purposes in BC.
